# Addition of nocturnal pollinators modifies the structure of pollination networks

**DOI:** 10.1038/s41598-023-49944-y

**Published:** 2024-01-12

**Authors:** Yedra García, Luis Giménez-Benavides, José M. Iriondo, Carlos Lara-Romero, Marcos Méndez, Javier Morente-López, Silvia Santamaría

**Affiliations:** 1 Area of Biodiversity and Conservation, ESCET, Universidad Rey Juan Carlos, C/Tulipán s/n, Móstoles, E-28933 Madrid, Spain; 2 Department of Biology, Lund University, SE-223 62 Lund, Sweden; 3 Island Ecology and Evolution Research Group, Instituto de Productos Naturales y Agrobiología (IPNA-CSIC), Avda. Astrofísico Francisco Sánchez 3, E-38206 San Cristóbal de La Laguna, Santa Cruz de Tenerife Spain

**Keywords:** Ecological networks, Community ecology

## Abstract

Although the ecological network approach has substantially contributed to the study of plant-pollinator interactions, current understanding of their functional structure is biased towards diurnal pollinators. Nocturnal pollinators have been systematically ignored despite the publication of several studies that have tried to alleviate this diurnal bias. Here, we explored whether adding this neglected group of pollinators had a relevant effect on the overall architecture of three high mountain plant-pollinator networks. Including nocturnal moth pollinators modified network properties by decreasing total connectivity, connectance, nestedness and robustness to plant extinction; and increasing web asymmetry and modularity. Nocturnal moths were not preferentially connected to the most linked plants of the networks, and they were grouped into a specific “night” module in only one of the three networks. Our results indicate that ignoring the nocturnal component of plant-pollinator networks may cause changes in network properties different from those expected from random undersampling of diurnal pollinators. Consequently, the neglect of nocturnal interactions may provide a distorted view of the structure of plant-pollinator networks with relevant implications for conservation assessments.

## Introduction

The ecological network approach has entailed a remarkable advance in the study of mutualistic plant-pollinator interactions^1–3^. Among other topics, ecological networks have been used to assess the consequences of habitat fragmentation and disturbance^4,5^, the impact of alien plant invasions^6,7^ or in the conservation of endangered plants^8^.

Adaptations to nocturnal pollination are widespread among flowering plants^9^. Yet, network studies of plant-pollinator interactions have paid little attention to nocturnal pollinators, except in a few noteworthy papers^7,10–13^. Nocturnal pollinators include insects (beetles^14^, bees^15^, moths^16,17^), as well as vertebrates (bats^18^, rodents^19^, other micromammals^20^). While some of these nocturnal pollinators (e.g., bats) may be of limited geographical or taxonomic importance^18^, others are very widespread. In particular, moths are spread worldwide^16,17^ and undoubtedly the most diversified group of nocturnal pollinators; just the two largest families of macro-moths (Macroheterocera) are more diverse than all Papilionoidea (Noctuidae and Geometridae, ca. 35,000 and 21,000 species, respectively)^21^. Therefore, leaving nocturnal moths out of plant-pollinator networks neglects a huge component of the architecture of biodiversity.

Building accurate ecological networks is crucial to properly understand the structure and dynamics of complex ecological systems^3,22,23^. Mutualistic networks based exclusively on diurnal flower visitors violate two fundamental requirements of community studies: sampling must be designed to avoid temporal bias and to achieve taxonomic independence^23^. In the case of plant-pollinator networks, taxonomic and temporal constrictions are unavoidably linked, because most nocturnal insects visiting flowers belong to exclusively night-active taxa. Thus, neglect of nocturnal moths could severely influence fundamental properties of networks such as nestedness, modularity and phylogenetic structure, derived properties such as robustness to extinctions, and their implications for conservation and restoration of ecosystem services^10,12,24^. Two alternative scenarios are conceivable when considering nocturnal moths in plant-pollinator networks^12^. First, nocturnal moths could be connected to the most linked plants of the network by preferential attachment. In this scenario, pollinators are more likely to interact with plants already visited by many species, potentially because they are more abundant, provide better resources, or are more attractive^25,26^. This scenario likely causes no major changes in network structure, besides increased network dimension and nestedness. Alternatively, nocturnal moths may adjust to the traditional concept of pollination syndromes, in which nocturnal moths should preferentially visit phalaenophilous plants -those with tubular white flowers and nocturnal floral anthesis, nectar secretion and odour emission at dusk or night^27^. In this latter scenario, nocturnal moths may conform distinct modules^28^ within the combined network (i.e., diurnal and nocturnal visits), which may increase modularity and decrease network nestedness.

To date only a few works have considered nocturnal pollinators in mutualistic networks, either alone^13,29–31^ or in combination with diurnal pollinators^7,10–12^. Several of these studies reported that some nocturnal pollinators formed specific modules^7,12^, but other nocturnal pollinators were part of mixed modules^12^. Devoto et al*.*^10^ reported similar properties of nocturnal and combined networks, but they did not perform a comparison between them. In sum, previous research highlights the important but overlooked role that nocturnal pollinators may have in pollination networks, and the complementarity between diurnal and nocturnal pollinators. However, to date, no formal comparison of the extent to which adding nocturnal pollinators to diurnal networks modifies network structure has been performed.

Here, we assemble the combined plant-pollinator networks from three high-mountain sites located in the Iberian Peninsula to assess the changes in network properties when nocturnal moths are considered. Studying plant-pollinator networks in high-mountain environments is relevant because they are key for preserving the functionality of these fragile ecosystems^32^. We address the following specific questions: (1) Do nocturnal moths preferentially interact with phalenophilous plants or do they visit the most linked plants in the network by preferential attachment? and (2) Are general network properties modified by the addition of the nocturnal moths?

## Methods

### Study sites

**Figure 1:**
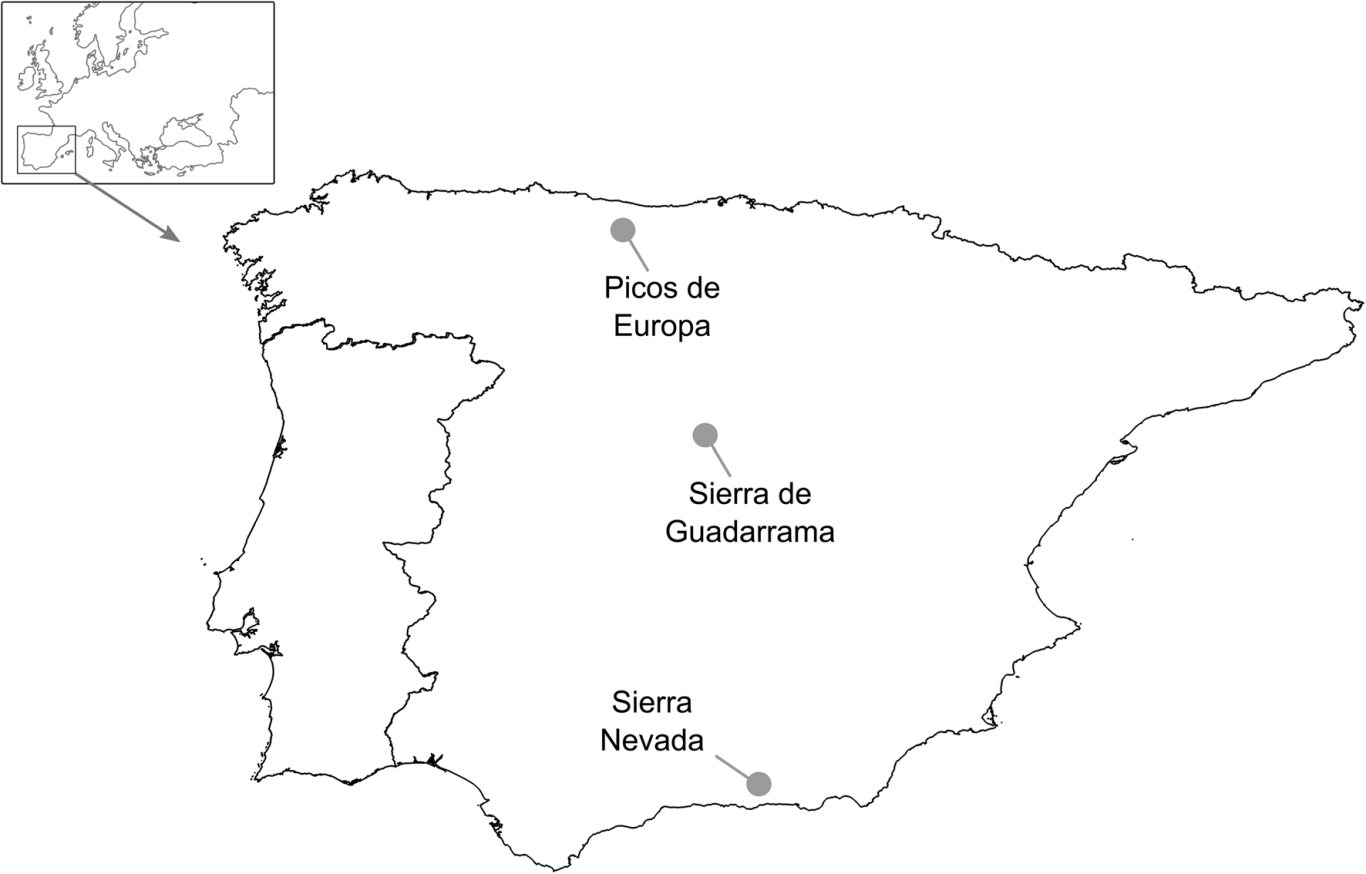
Study sites. Spatial locations of the three sample sites in the Iberian Peninsula.

Three typical high mountain plant communities were chosen along a latitudinal and climatic gradient in the Iberian Peninsula: Picos de Europa (N Spain, Atlantic climate, 2050 m a.s.l.), Sierra de Guadarrama (central Spain, continental Mediterranean climate, 2210 m a.s.l.) and Sierra Nevada (S Spain, Mediterranean climate, 2850 m a.s.l.) (Fig. 1). These sites represented equivalent altitudinal vegetation belts above treeline, although their absolute elevation differed due to the contrasting climatic conditions of the three mountain ranges (see Santamaría et al.^32^ and Lara-Romero et al*.*^33^ for further details).

### Sampling protocol

Diurnal and nocturnal plant-flower visitor networks (hereafter, plant-pollinator networks) were built for each site during the flowering season of 2010 (Picos de Europa) and 2011 (Sierra de Guadarrama and Sierra Nevada). To build the diurnal networks, interactions between plants and floral visitors were recorded along diurnal transects at each site, where all insects contacting the reproductive structures of the flowers were recorded. The sampled area differed between sites from 500 × 250 m in Picos de Europa to 150 × 100 m in Sierra Nevada and 100 × 60 m in Sierra de Guadarrama. These differences were dependent on the small-scale heterogeneity of vegetation. The transects were evenly distributed throughout the study area. The length of the transects varied depending on the size of the study area. Diurnal transects were performed from 10 to 18 h on sunny days with mild wind conditions for pollinator activity. Species vouchers were captured and identified to species or morphospecies level. Sampling involved 2–6 people during 5 to 7 weeks from June to August for a total of 9679, 3278 and 11,754 recorded visits in Picos de Europa, Sierra de Guadarrama and Sierra Nevada, respectively (see Santamaría et al*.*^32^ and Lara-Romero et al*.*^33^ for further details).

Nocturnal plant-pollinator networks were built for each site by trapping moths using light traps and analysing their pollen loads. Light traps consisted of a UV light surrounded by three white triangular sheets. Moths landing on the sheets were immediately trapped and stored in individual vials with a small piece of tissue and some drops of ethyl acetate. This procedure was essential to avoid pollen loss or pollen transfer among individuals, thus allowing a reliable estimation of plant-moth interactions and pollen loads. Three (four in Sierra de Guadarrama) trapping sessions were carried out along the flowering period, about one week apart and around the flowering peak. The sampling period each night was from dusk to about 01:00 am (ca. 3–3.5 h). To minimize the intrinsic limitations of light traps, such as the attraction of moths from relatively large distances or variation in their attraction ability to different species^10,34,35^, traps were located at the central area of each study site.

### Pollen extraction and identification

For identification purposes, a pollen reference collection was compiled at each site. Flowers of each entomophilous plant species were harvested and pollen was collected, stained with basic fuchsine and fixed in microscope slides^36^. Pollen pictures were taken with a reflex camera (Canon 450D) coupled to a phase contrast microscope (Olympus Bx51). To build a reference pollen key, pollen size and ornamentation for each plant species was recorded by using ImageJ^37^.

Moths were mounted and pollen loads were collected by rubbing small fuchsine jelly cubes around the head and mouthparts^36^. Cubes were melted and mounted on slides, and pollen grains were counted in the microscope. Then, the pollen grains were compared to the pollen reference key and identified to species. The only exception were two closely related *Sedum* species with indistinguishable pollen grains, that were classified as the same morphospecies^10^ (see Table S2). To avoid a potential bias by heterospecific pollen transport^10^, an interaction was only scored when an individual nocturnal moth carried three or more pollen grains of that particular plant species.

### Data analysis

We assembled three qualitative (i.e., presence-absence) interaction networks per site: one considering exclusively diurnal visits (hereafter, diurnal network), one considering exclusively nocturnal visits (hereafter, nocturnal network) and one considering both diurnal and nocturnal visits (hereafter combined network). Assembly of all networks was qualitative to avoid the difficulties in comparing quantitative interactions obtained with different sampling methodologies^12^ (see also Discussion “Caveats and further developments” section). Interaction and species sampling completeness for diurnal and nocturnal networks were calculated following Chacoff et al*.*^38^ with the R-package vegan version 2.4–5^39^. To obtain the expected asymptotic richness of species and interactions, this method uses the non-parametric Chao 2 estimator that is particularly appropriate for small sample sizes^38,40^.

We assessed whether nocturnal moths preferentially attached to the plants already showing the highest number of links in the diurnal network, by performing a *t*-test that compared differences in the diurnal degree (number of links) rank between plants with and without nocturnal moths. In the case of a tie, the average rank was assigned to the plant species involved.

Fifteen network properties of diurnal and combined networks (Table 1) were assessed using the R-packages bipartite version 2.08^41^ and vegan version 2.4–5^39^. Pollinator, plant, and total nestedness were measured using NODF^42^. Bipartite modularity (*Q*) and number of modules were estimated using the DIRTLPAwb+algorithm^43^. In the combined networks, module composition was checked to identify the existence of modules consisting only of nocturnal moths. To assess the significance of NODF and Q we used Z-test against a fixed–fixed null distribution derived from 500 random networks (for NODF) and 100 networks (for Q) with the same number of plants, pollinators and interactions as the observed networks. The estimation of network robustness was based on species extinction curves, in which the proportion of "secondary extinctions" caused by the accumulation of random "primary extinctions" among their mutualistic partners is represented^44^. We used the function *second.extinct* in the bipartite package in R^41^ to simulate species extinction curves, averaging from 100 repetitions. Then, we calculated two values for each network: (i) robustness to pollinator extinction (R_50_ A), i.e., the minimum fraction of primary extinctions of pollinators that causes ≥ 50% of secondary extinction of plants and (ii) robustness to plant extinction (R_50_ P), i.e., the minimum fraction of primary extinctions of plants that causes ≥ 50% of secondary extinction of pollinators^45,46^. We then calculated the percentage change in all these network descriptors after adding the nocturnal interactions to the diurnal networks (Table 1).

**Table 1:** Properties of the diurnal (D) and combined (C: diurnal plus nocturnal) networks.

Network property	Picos de Europa			Sierra de Guadarrama			Sierra Nevada		
	D	C	Obs. (%)	D	C	Obs. (%)	D	C	Obs. (%)
No. of animals (A)	120	136	11.8	102	116	12.0	115	128	10.2
No. of plants (P)	92	95	32.	17	17	0	32	34	5.9
Matrix size (A x P)	11,040	12,920	14.5	1734	1972	12.1	3712	4352	14.7
No. of interactions (i)	1136	1158	1.9	315	349	9.7	543	563	3.6
Web asymmetry	0.132	0.177	25.4	0.714	0.742	3.8	0.565	0.580	2.6
Connectivity A (i/A)	9.467	8.515	− 11.1	3.088	3.009	− 2.6	4.722	4.398	− 7.3
Connectivity P (i/P)	12.348	12.189	− 1.3	18.529	20.529	9.7	16.969	16.559	− 2.5
Connectivity total (i/[A + P])	5.358	5.013	− 6.9	2.647	2.644	− 0.1	3.694	3.475	− 6.3
Connectance (i/[A x P])	0.103	0.090	− 14.4	0.182	0.179	− 1.7	0.148	0.129	− 14.7
NODF	37.053	32.929	− 12.5	36.410	33.739	− 7.9	40.205	34.337	− 17.1
NODF A	32.090	28.292	− 13.4	36.058	33.500	− 7.6	39.370	33.752	− 16.7
NODF P	45.518	42.461	− 7.2	49.746	45.462	− 9.4	51.243	42.803	− 19.7
Modularity Q	0.267	0.279	4.3	0.324	0.323	− 0.3	0.275	0.281	2.1
Robustness (R_50_ A)	0.925	0.926	0.1	0.951	0.957	− 0.6	0.948	0.953	0.5
Robustness (R_50_ P)	0.880	0.842	− 4.5	0.7065	0.706	− 8.4	0.781	0.765	− 2.1

“P” denotes plant species, “A” denotes animal species and “i” denotes interactions in the networks, “Obs. (%)” is the percentage of change when nocturnal pollinators are added to the diurnal network.

**Figure 2:**
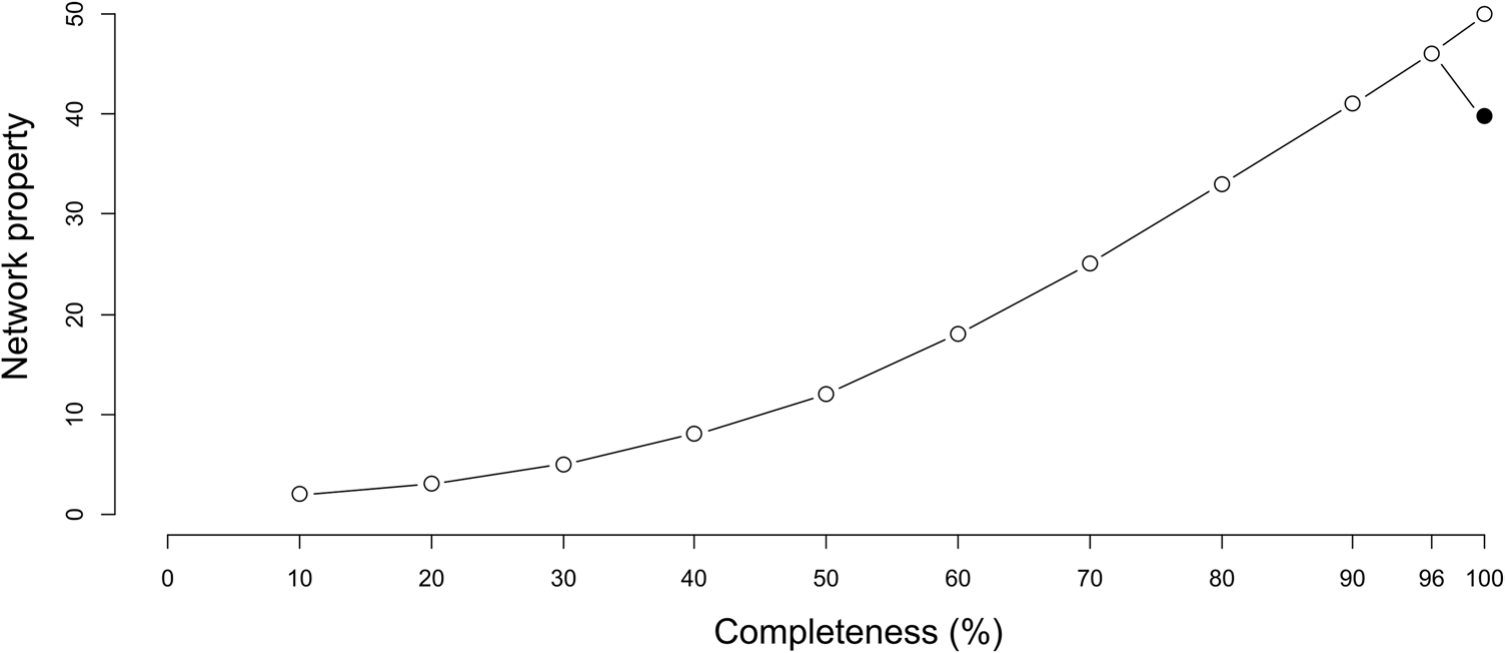
Testing for changes in network properties. Hypothetical example of the change in a network property as new interactions are added. Grey dots represent the trend of the network property as a function of the percentage of diurnal interactions added. The black dot represents the addition of nocturnal instead of diurnal interactions. The bifurcation at 100% sampling completeness shows the expected break produced when adding nocturnal instead of diurnal interactions. Notice that in this example the number of nocturnal interactions added represents 4% of the number of total diurnal interactions recorded. Because of this, the bifurcation point is drawn at 96% completeness.

To address whether the network structure was modified by the addition of the nocturnal moths or whether the lack of these nocturnal pollinators could be simply considered a case of undersampling (i.e., it is equivalent to improve the sampling of diurnal networks), we focused on eight network properties (Table 2). We assessed how these properties were affected when a random set of diurnal pollinators was substituted by a set of nocturnal moths using an approach inspired in how data resampling influences network properties^47,48^. Assuming that *n* is the number of nocturnal interactions and *d* is the number of diurnal interactions, we randomly subsampled the diurnal network starting from 10% of diurnal interactions and subsequently adding sets of 10% of interactions until we reached *d*-*n* interactions (Fig. 2). Each random subsampling was replicated 100 times and the average value and the confidence intervals for each network property were calculated at each subsampling level. This gradient of subsampling ended with the total diurnal network, which was compared to an alternative network (100 random replicates) with *d* interactions consisting of the *n* nocturnal interactions added to the subsampling with *d-n* interactions. This comparison aimed to discern any disparities in network properties when introducing *n* diurnal interactions versus *n* nocturnal interactions to a network characterized by *d*-*n* interactions. Our expectation was that if the presence of nocturnal moths modified network properties, deviations from the trends observed in the subsampled diurnal network would become evident (as depicted in Fig. 2). To ascertain the significance of these deviations, we considered a departure to be significant when the confidence interval of a network metric value for the resampled diurnal network, which encompasses nocturnal interactions (black dot in Fig. 2), did not overlap with the equivalent value for the complete diurnal network (last grey dot in Fig. 2).

**Table 2:** Comparison of network descriptors and robustness for the diurnal network with 100% completeness and the resampled diurnal and nocturnal network.

	Picos de Europa		Sierra de Guadarrama		Sierra Nevada	
	Diurnal	Diurnal+Nocturnal	Diurnal	Diurnal+Nocturnal	Diurnal	Diurnal+Nocturnal
Connectance	0.103	0.087 (0.087, 0.087)	0.182	0.167 (0.167, 0.168)	0.148	0.127 (0.126, 0.127)
Web asymmetry	0.132	0.171 (0.171, 0.172)	0.714	0.734 (0.733, 0.735)	0.565	0.575 (0.574, 0.576)
Connectivity total (i/[A + P])	5.358	4.907 (4.905, 4.910)	2.647	2.466 (2.459, 2.472)	3.694	3.393 (3.388, 3.398)
NODF total	37.053	32.370 (32.328, 32.411)	36.410	31.604 (31.453, 31.756)	40.205	33.362 (33.258, 33.466)
Modularity Q	0.267	0.281 (0.281, 0.282)	0.324	0.343 (0.341, 0.345)	0.275	0.290 (0.289, 0.291)
R_50_ A	0.925	0.923 (0.922, 0.924)	0.951	0.955 (0.955, 0.956)	0.948	0.947 (0.946, 0.948)
R_50_ P	0.880	0.846 (0.845, 0.848)	0.765	0.707 (0.705, 0.709)	0.781	0.762 (0.760, 0.764)

The latter was constructed by extracting *n* diurnal interactions and adding *n* nocturnal interactions to the diurnal network, where *n* represents the total number of nocturnal interactions sampled in each study site. The resampled diurnal and nocturnal network depicts, for each network descriptor, the average value and the 95% confidence intervals. “P” denotes plant species, “A” denotes animal species and “i” denotes interactions in the networks.

## Results

A total of 132 nocturnal moths (Picos de Europa), 168 (Sierra de Guadarrama) and 118 (Sierra Nevada) were captured. Three or more pollen grains were found in 20%, 29% and 15% of the moths. Overall, nocturnal moths interacted with 33 plant species and four of the latter only showed nocturnal interactions (see Appendix S1 and S2 in Supporting Information). To our knowledge, we provide the first evidence of interactions with nocturnal moths for Gentianaceae and Plantaginaceae. Nocturnal networks were considerably smaller than diurnal networks, comprising 13–16 moth species, 10–21 plant species, and 20–34 interactions, with matrix sizes ranging from 208 to 680 (Appendix S1 in Supplementary Information). Diurnal networks comprised 102–120 animal species, 17–92 plant species, 315–1136 interactions, and had matrix sizes ranging from 1734 to 11,040 (Table 1).

**Figure 3:**
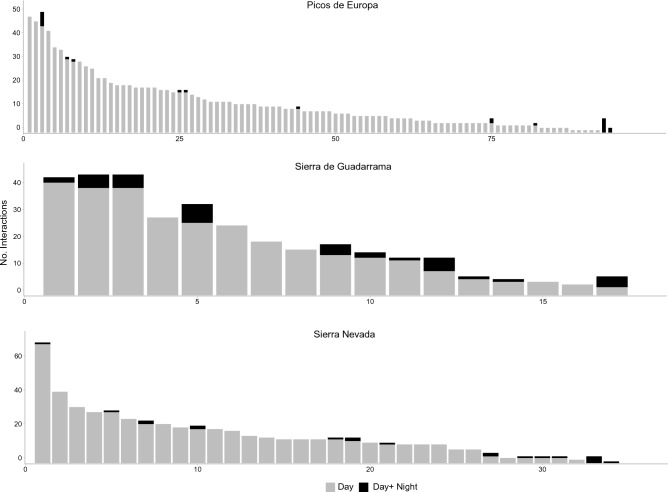
Number of diurnal and nocturnal interactions per plant species. Number of interactions of the different plant species in the diurnal networks (grey bars) and number of interactions added by nocturnal moths (black bars).

Eighty-five per cent of the plant species visited by nocturnal moths showed a diurnal syndrome. In Sierra de Guadarrama and Sierra Nevada, eleven plant species attracted both diurnal and nocturnal pollinators, whereas in Picos de Europa only eight plant species did (Tables S1, S2, S3 and Appendix S1 in the Supplementary Information). No significant differences in diurnal degree rank were found between the plants that interacted with nocturnal moths and those with only diurnal visits in any of the sites (Picos de Europa: *t*_93_ = − 1.915, *P* = 0.742; Sierra de Guadarrama: *t*_16_ = − 0.195, *P* = 0.848; Sierra Nevada: *t*_33_ = − 1.311, *P* = 0.199). Nocturnal moths interacted with plants of very different degree, from highly to scarcely connected and even with plants with no diurnal visits (Fig. 3 and Appendix S2 in Supplementary Information).

The combined networks showed higher asymmetry and modularity than diurnal networks, with a few exceptions, including the modularity in the combined network from Guadarrama (Table 1). Diurnal and combined networks were significantly modular compared to random networks (*Z*-test: all *P* < 0.01). Nocturnal moths and the plants visited by them were not grouped in specific modules except in Picos de Europa (see Figure S1of Appendix S2 in Supplementary Information). In Sierra Nevada, the addition of nocturnal moths increased the number of modules from five to seven. Both diurnal and combined networks were significantly nested (*Z*-test: all *P* < 0.01) in all study sites, excepting the diurnal network in Picos de Europa (*P* = 0.205). Combined networks showed lower nestedness, connectivity for pollinators, connectivity for plants (except in Sierra de Guadarrama) as well as lower total connectivity and connectance (Table 1).

**Figure 4:**
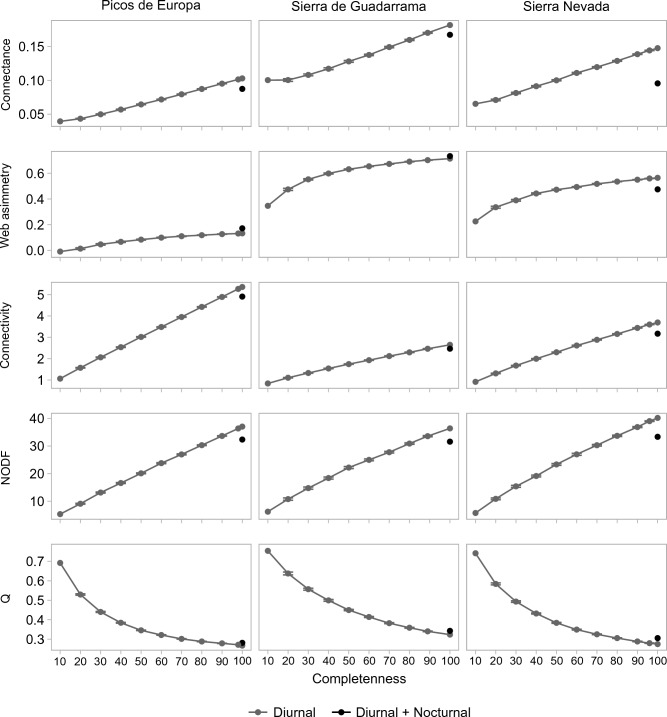
Subsampling network properties. Subsampling performed for each network property. The dots indicate the mean values, and the error bars the 95% confidence intervals. In some cases, the width of the dot is larger than the error bars. The grey line indicates the different subsamples of the diurnal networks. The black circle indicates the addition of the *n* nocturnal interactions to the resampled diurnal network, built by removing *n* diurnal interactions from the *d* diurnal interactions and adding the *n* nocturnal interactions. Notice that the subsampling *d*-*n* differs among sites (98% for Picos de Europa, 96% for Sierra Nevada, 89% for Sierra de Guadarrama) due to different size of the nocturnal network.

**Figure 5:**
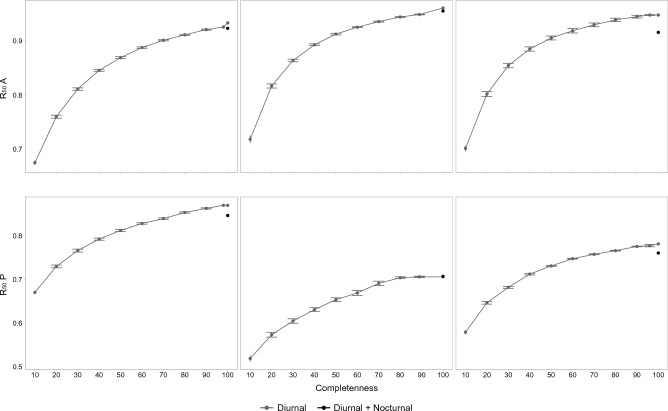
Subsampling robustness. Subsampling performed for robustness to the extinction of animals (R_50_ A) and robustness to the extinction of plants (R_50_ P). The dots indicate the mean values, and the error bars the 95% confidence intervals. In some cases, the width of the dot is larger than the error bars. The grey line indicates the trend of the different subsamples of the diurnal networks. The grey dots depict the robustness values corresponding to the resampled diurnal networks. The black dots indicate the robustness value after the addition of the *n* nocturnal interactions to the resampled diurnal network with *d-n* diurnal interations. Notice that the subsampling *d*-*n* differs among sites (98% for Picos de Europa, 96% for Sierra Nevada, 88% for Sierra de Guadarrama) due to different sizes of the nocturnal networks.

The resampling of the diurnal network indicated a gradual increase in connectance, web asymmetry, NODF, connectivity and robustness (Figs. 4, 5), as well as a gradual decrease in modularity (Fig. 4). Against these general trends, replacing *n* diurnal interactions by *n* nocturnal interactions entailed a significant break in the trend of all network properties (Figs. 4, 5; Table 2). Connectance, NODF, connectivity, robustness to the extinction of plant species decreased after adding the nocturnal interactions, while asymmetry and modularity increased (Figs. 4, 5; Table 2). Robustness to the extinction of pollinator species differed among networks (Figs. 4, 5; Table 2). It decreased in Picos de Europa, increased in Sierra de Guadarrama and did not differ significantly in Sierra Nevada (Figs. 4, 5; Table 2).

## Discussion

The addition of nocturnal moths had a relevant effect on the overall architecture of the three high mountain plant-pollinator networks. Moths modified network properties by decreasing connectance, nestedness, connectivity and robustness to plant extinction and by increasing web asymmetry and modularity. Our results indicate that disregarding the nocturnal component of plant-pollinator networks may cause changes in network properties different from those expected from random undersampling of diurnal pollinators and lead to a misinterpretation of plant-pollinator networks. It is remarkable that the addition of this nocturnal component did not conform well to any of the two expected scenarios: nocturnal moth pollinators were not preferentially connected to the most linked plants of the network and were grouped into a single nocturnal module only in one network. These results highlight the potential consequences of underestimating the role of nocturnal moths as pollinators in natural ecosystems.

### Nocturnal moths visited a random sample of plant species in most networks

None of the three networks studied showed preferential attachment of nocturnal moths to the most linked plants. Only in one network, moths met the expectations of the pollination syndrome concept and conformed to a particular nocturnal module. Thus, these results did not adjust to any of the two initially set scenarios. In networks with a heterogeneous distribution of links per species, new randomly recorded species are assumed to preferentially attach to the most linked species^25,28^ but this is not always true^49^. Syndrome-related modules have been found in several mutualistic networks^44,50^ including two plant-pollinator networks in which both diurnal and nocturnal pollinators have been included^7,12^. Nevertheless, the extent to which network modules match plant pollination syndromes is variable^12^, and increases with increasing specialization of the interactions^51,52^. In this study, the absence of a nocturnal module in two out of three networks is unsurprising because generalist pollination interactions are expected to be the rule in harsh and variable environments such as high mountain ecosystems^53,54^.

Our study warns against a naïve inference of pollinators from floral traits. A small, but not trivial, number of plant species showed a large mismatch between expected and actual pollinators. For example, plants with expected nocturnal moth pollination, such as *Silene boryi* and *S. ciliata*, were visited both by diurnal and nocturnal insects (Appendix S1). Diurnal visitation of species with phalaenophilous syndrome are well known in *Silene*^55,56^ and, more generally, have been reported in desert^57^, temperate^58,59^ and tropical ecosystems^12^. For instance, in a plant-pollinator network from the Neotropics, flowers with nocturnal anthesis that remained open during the day were important connectors of the diurnal and nocturnal components^12^. More interestingly, some plant species apparently adapted to diurnal pollinators were also visited by nocturnal moths^13,60^. The most striking case were *Linaria* species, for which a bee, bee-fly and butterfly syndrome had been described^61^. These results, together with those of previous nocturnal networks^7,10,11,29^, are unveiling overlooked nocturnal visitors for many flowering plants and call for future work to determine the contribution of nocturnal pollinators to plant reproduction. This invites a reconsideration of currently accepted levels of plant specialization^62^.

### Addition of nocturnal moths modified network properties, including modularity and robustness

The addition of a moderate number of interactions and species of nocturnal moths resulted in changes in most of the analysed network properties. Some network studies have previously targeted neglected groups nocturnal pollinators^7,10,12,13,29^. For instance, Walton et al.^13^ detected a higher complexity (higher linkage density and interaction diversity) in the nocturnal pollination network than in the diurnal networks in an agro-ecosystem. However, this is the first study assessing in a comprehensive way the differences in structural properties of networks with and without neglected groups of pollinators. Given the absence of previous studies that follow a similar approach, we decided to compare these results with the general trends obtained in studies testing subsampling effects on network properties. In general, the values of all metrics increase with increasing sampling effort, except for binary modularity and connectance that decrease^47,63^ (but see Rivera-Hutinel et al.^64^). Our results strikingly departed from these trends in two ways. First, the addition of nocturnal moth pollinators led to opposite changes in trend for modularity and nestedness to those reported for subsampling. Second, the magnitude of the changes in most properties was higher than the usually reported for subsampling^47,63,64^. This suggests that adding nocturnal pollinators (1) has consequences on network connectance that cascade to other network properties and (2) is not equivalent to better sampling of diurnal networks.

Current wisdom is that mutualistic networks are robust^25,65,66^ and that robustness is reliably assessed in incompletely sampled networks^64^. However, our results indicate that neglecting nocturnal moths can lead to an overestimation of network robustness. In evolutionary terms, this adds complexity to the arguments for the evolution of generalized pollination systems^38,67,68^.

### Caveats and further developments

Our results highlight the importance of including nocturnal pollinators in plant-pollinator networks. Ideally, nocturnal pollinators should be added using the same sampling methods as those used for diurnal networks. While this is, in principle, feasible, exceptions may arise, such as when integrating different studies into a single plant-pollinator network^12^.The main potential caveat of combining diurnal and nocturnal networks obtained using different methods is that it can lead to biases. Certainly, comparisons of visit- and pollen-based networks indicate that pollen-transport networks are smaller and more specialized compared with their respective visitation networks^13,69–71^ (but see Jędrzejewska-Szmek and Zych^72^ and Walton et al*.*^13^). However, in terms of network metrics such as nestedness, modularity, and connectance (evaluated in this study), the results of these comparisons lack clear patterns^72–76^. This diversity of results aligns better with “noise” than with a consistent bias due to differences in sampling methods. On the other hand, the few existing studies combining pollen and flower visitor networks^12,73,75^ have shown higher connectivity and nestedness compared to visitor-only networks^73,75^. If the addition of nocturnal interactions would simply introduce a bias in network parameters, we would expect the combined network parameters to be biased in the same direction shown by the comparative studies of visit vs. pollen networks. However, our results show a change in the opposite direction. This allows us to be confident that our results are unbiased with respect to the sampling protocols used. Nevertheless, we acknowledge that combining networks constructed using different sampling techniques entails interpretation challenges and we encourage further studies to assess the generality of our findings.

A second caveat, inherent to any sample methodology, could be sample completeness. Interaction and species sampling completeness were lower in the nocturnal networks, compared to the diurnal ones, especially for interactions (Table S4 of Appendix S3 in Supplementary Information). In any case, species sampling completeness of the combined networks revealed that, on average, more than 50% of both plant and pollinators could be detected for all the study sites (Table S5 of Appendix S3 in Supplementary Information), which compares favorably with the only estimate of species completeness performed for nocturnal networks^11^. Species completeness was greater than interaction completeness in the nocturnal networks, as shown by previous studies on pollination and dispersal networks^38,77^. This lower interaction completeness in the nocturnal networks (especially in Sierra Nevada) may respond to several factors such as a low sampling effort and a possible inflated expected richness computed by the Chao 2 estimator, that considers singletons and doubletons to estimate the number of undetected interactions^38,40^. This may be particularly important in the nocturnal networks studied, in which most moths were rare (they fell in the light traps only once) and they bore pollen from one or two plant species. Although we could expect small network sizes for the nocturnal side of high mountain pollination networks and in turn low sampling efforts, only further research will reveal the actual frequency of nocturnal interactions. Recently, a multi-level approach has been used to study diurnal and nocturnal networks^12^. Here, we suggest exploring the change in network properties by subsampling of the diurnal network with the addition of the nocturnal network. In sum, these results call for new studies combining diurnal and nocturnal pollination by integrating analysis approaches that consider different sampling efforts.

Building nocturnal plant-pollinator networks is challenging. Based on our experience and previous works on nocturnal moth species inventories, we can draw some methodological advice. (1) For a complete assessment of species diversity, at least 5–10 days of sampling will yield high percentages of the expected species (e.g., Beck and Linsenmair^34^). (2) Immediate hand-sampling at the light source and careful individual packing are necessary to avoid pollen contamination among specimens. (3) Although it is usually assumed that moth visits to flowers are particularly concentrated on twilight and first night hours, light traps should be ideally set during the whole night because shorter sessions could miss species with different flight times^34^. (4) As with the sampling of diurnal pollination networks^78,79^, nocturnal sampling should be conducted throughout the flowering season, especially in ecosystems with high seasonality. (5) Nocturnal moths from different families may be differently attracted to light^35^, and thus the combination of light traps with other sampling techniques like bait traps may be appropriate^80^.

In a more applied perspective, combined networks will provide fundamental information about the role of nocturnal pollinators^10,12,29^ and will contribute to assess the effects of increasing threats that affect this group, such as increasing light pollution^11,16,81^. Ultimately, these threats may jeopardize ecosystem services provided by nocturnal pollinators by disrupting their interactions with plants^11,16^. The present study indicates that ignoring nocturnal pollinators leads to an underestimation of functional and phylogenetic diversity. Since plant diversity closely depends on functional diversity of pollinators^82^, information on the dynamics of nocturnal moth assemblages and their role on plant-pollinator networks structure is crucial for a reliable monitoring of the conservation status of plant communities^17,81^. Consequently, neglect of nocturnal interactions may provide a distorted view of the structure of pollination networks.

### Supplementary Information

Supplementary Information.

## Data Availability

The data used on this research are openly available at the following Zenodo link: https://zenodo.org/records/10391505.
