# Supplementary material for: Addition of nocturnal pollinators modifies the structure of pollination networks
Source: Sci Rep. 2024 Jan 12;14:1226. doi: 10.1038/s41598-023-49944-y (PMC10786900; doi:10.1038/s41598-023-49944-y)
Supplement: Supplementary file 1 — Supplementary Information. [file 41598_2023_49944_MOESM1_ESM.docx]

Appendix S1. List of plant and moth species involved in the nocturnal networks at each study site.

**Table S1.** Plant and moth species from Picos de Europa nocturnal network, type of expected pollinators (diurnal or nocturnal) of the plant species based on main floral traits (corolla shape and colour, timing of scent emission when available) and number of interactions on the diurnal (D) and the nocturnal (N) networks. Plant and moth species are listed in alphabetical order. Asterisks denote plant species for which we report the first evidence of interactions with nocturnal moth visitors (some of them, previously shared for the review by Gómez et al., 2016).

| Plant species | Family | Expected pollinators | Interactions | |
| --- | --- | --- | --- | --- |
|  |  |  | D | N |
| *Androsace villosa* L.* | Primulaceae | Diurnal | 10 | 1 |
| *Anthyllis vulneraria* ssp. *pyrenaica* Cullen* | Fabaceae | Diurnal | 17 | 1 |
| *Arabis ciliata* Clairv. | Brassicaceae | Diurnal | 3 | 1 |
| *Eryngium bourgatii* Gouan* | Apiaceae | Diurnal | 30 | 1 |
| *Linaria supina* (L.) Chaz.* | Plantaginaceae | Diurnal | 4 | 2 |
| *Minuartia verna* (L.) Hiern* | Caryophyllaceae | Diurnal | 17 | 1 |
| *Pritzelago alpina* ssp. *alpina* (Sievers) Kuntze | Brassicaceae | Diurnal | 31 | 1 |
| *Silene acaulis* (L.) Jacq. | Caryophyllaceae | Diurnal/Nocturnal | 45 | 6 |
| *Silene ciliata* Pourr. | Caryophyllaceae | Nocturnal | 0 | 6 |
| *Silene vulgaris* ssp*. glareosa* (Jord.) Mardsen-Jones & Turrill | Caryophyllaceae | Nocturnal | 0 | 2 |

**Table S1** (Continued).

| Moth species | Family | Expected visitors | Interactions | |
| --- | --- | --- | --- | --- |
|  |  |  | D | N |
| *Agrotis simplonia* Geyer | Noctuidae | _ | 0 | 2 |
| *Apamea furva* Den & Schiff. | Noctuidae | _ | 0 | 2 |
| *Apamea platinea* Treitschke | Noctuidae | _ | 0 | 1 |
| *Apamea zeta* Treitschke | Noctuidae | _ | 0 | 3 |
| *Entephria nobiliaria* H-Sch. | Geometridae | _ | 0 | 1 |
| Geometridae spp. | Geometridae | _ | 0 | 2 |
| *Gnophos obfuscatus* Den. & Schiff. | Geometridae | _ | 0 | 1 |
| *Hadena ruetimayeri* Boursin | Noctuidae | _ | 0 | 1 |
| Pyraloidea 2 | SupF. Pyraloidea | _ | 0 | 1 |
| *Rheumaptera* sp. | Geometridae | _ | 0 | 1 |
| *Rhyacia simulans* Hufnagel | Noctuidae | _ | 0 | 1 |
| *Setina cantabrica* Freina & Witt | Arctiidae | _ | 0 | 1 |
| *Standfussiana dalmata* Stgr. | Noctuidae | _ | 0 | 1 |
| *Xestia ashworthii* Doubleday | Noctuidae | _ | 0 | 1 |
| Morphospecies 1 | Unknown | _ | 0 | 1 |
| Morphospecies 3 | Unknown | _ | 0 | 1 |

**Table S2.** Plant and moth species from Guadarrama nocturnal network, type of expected pollinators (diurnal or nocturnal) of the plant species based on main floral traits (corolla shape and colour, timing of scent emission when available) and number of interactions on the diurnal (D) and the nocturnal (N) networks. Plant and moth species are listed in alphabetical order. Asterisks denote plant species for which we report the first evidence of interactions with nocturnal moth visitors.

| Plant species | Family | Expected pollinators | Interactions | |
| --- | --- | --- | --- | --- |
|  |  |  | D | N |
| *Adenocarpus hispanicus* (Lam.) DC.* | Fabaceae | Diurnal | 14 | 2 |
| *Biscutella intermedia* Gouan | Brassicaceae | Diurnal | 6 | 1 |
| *Cytisus oromediterraneus* Rivas Mart. et al.* | Fabaceae | Diurnal | 27 | 7 |
| *Euphrasia willkommii* Freyn* | Scrophulariaceae | Diurnal | 5 | 1 |
| *Gentiana lutea* L.* | Gentianaceae | Diurnal | 9 | 5 |
| *Jasione crispa* (Pourret) Samp.* | Campanulaceae | Diurnal | 40 | 5 |
| *Jurinea humilis* (Desf.) DC.* | Asteraceae | Diurnal | 42 | 2 |
| *Sedum* spp.* | Crassulaceae | Diurnal | 15 | 4 |
| *Senecio pyrenaicus* L.* | Asteraceae | Diurnal | 40 | 5 |
| *Silene ciliata* Pourr. | Caryophyllaceae | Nocturnal | 3 | 4 |
| *Solidago virgaurea* L. | Asteraceae | Diurnal | 13 | 1 |
| Moth species |  |  |  | |
| *Anania terrealis* Treitschke | Cambridae | _ | 0 | 1 |
| *Apamea monoglypha* Hfn. | Noctuidae | _ | 0 | 2 |
| *Autographa gamma* L. | Noctuidae | _ | 0 | 2 |
| *Calophasia hamifera* Staudinger | Noctuidae | _ | 0 | 1 |
| *Chersotis elegans* Eversmann | Noctuidae | _ | 0 | 1 |
| *Euxoa continentalis* Reisser | Noctuidae | _ | 0 | 6 |
| *Evergestis lupalis* Zerny | Cambridae | _ | 0 | 1 |
| *Hadena consparcatoides* Schawerda | Noctuidae | _ | 0 | 1 |
| *Lasyonicta proxima* Hübner | Noctuidae | _ | 0 | 8 |
| *Peridroma saucia* Hübner | Noctuidae | _ | 0 | 1 |
| Morphospecies 1 | Geometridae | _ | 0 | 6 |
| Morphospecies 2 | Geometridae | _ | 0 | 2 |
| Morphospecies 3 | Unknown | _ | 0 | 1 |
| Morphospecies 4 | Unknown | _ | 0 | 3 |

**Table S3.** Plant and moth species from Sierra Nevada nocturnal network, type of expected pollinators (diurnal or nocturnal) of the plant species based on main floral traits (corolla shape and colour, timing of scent emission when available) and number of interactions on the diurnal (D) and the nocturnal (N) networks. Plant and moth species are listed in alphabetical order. Asterisks denote plant species for which we report the first evidence of interactions with nocturnal moth visitors.

| Plant species | Family | Expected  pollinators | Interactions | |
| --- | --- | --- | --- | --- |
|  |  |  | D | N |
| *Arenaria tetraquetra* L.* | Caryophyllaceae | Diurnal | 70 | 1 |
| *Campanula herminii* Hoffmanns & Link* | Campanulaceae | Diurnal | 11 | 1 |
| *Carduus carlinoides* Gouan* | Asteraceae | Diurnal | 20 | 2 |
| *Cerastium cerastoides* (L.) Britt.* | Caryophyllaceae | Diurnal | 23 | 2 |
| *Galium pyrenaicum* Gouan* | Rubiaceae | Diurnal | 0 | 4 |
| *Hormathophylla spinosa* (L.) P. Küpfer | Brassicaceae | Diurnal | 3 | 2 |
| *Linaria aeruginea* (Gouan) Cav* | Plantaginaceae | Diurnal | 3 | 1 |
| *Linaria glacialis* Boiss.* | Plantaginaceae | Diurnal | 0 | 1 |
| *Lotus corniculatus* L.* | Fabaceae | Diurnal | 13 | 2 |
| *Ranunculus demissus* DC.* | Ranunculaceae | Diurnal | 30 | 1 |
| *Senecio boissieri* D.* | Ranunculaceae | Diurnal | 4 | 2 |
| *Silene boryi* Boiss. | Caryophyllaceae | Nocturnal | 3 | 1 |
| *Silene rupestris* L. | Caryophyllaceae | Diurnal | 14 | 1 |
| Moth species |  |  |  |  |
| *Apamea platinea* Eversmann | Noctuidae | _ | 0 | 3 |
| *Chersotis larixia* Guenée | Noctuidae | _ | 0 | 1 |
| *Hada plebeja* L. | Noctuidae | _ | 0 | 1 |
| *Pseudohadena halimi* Mill. | Noctuidae | _ | 0 | 1 |
| Morphospecies 1 | Unknown | _ | 0 | 1 |
| Morphospecies 2 | Unknown | _ | 0 | 3 |
| Morphospecies 3 | Unknown | _ | 0 | 1 |
| Morphospecies 4 | Unknown | _ | 0 | 1 |
| Morphospecies 5 | Unknown | _ | 0 | 1 |
| Morphospecies 6 | Unknown | _ | 0 | 3 |
| Morphospecies 9 | Unknown | _ | 0 | 1 |
| Morphospecies 11 | Unknown | _ | 0 | 1 |
| Morphospecies 12 | Unknown | _ | 0 | 1 |

**References**

Gómez, JM, Torices, R, Lorite, J, Klingenberg, CP & Perfectti, F. (2016). The role of pollinators in the evolution of corolla shape variation, disparity and integration in a highly diversified plant family with a conserved floral bauplan. *Ann. Bot.*, 117, 889-904.

Appendix S2. Combined qualitative interaction networks at each study site.


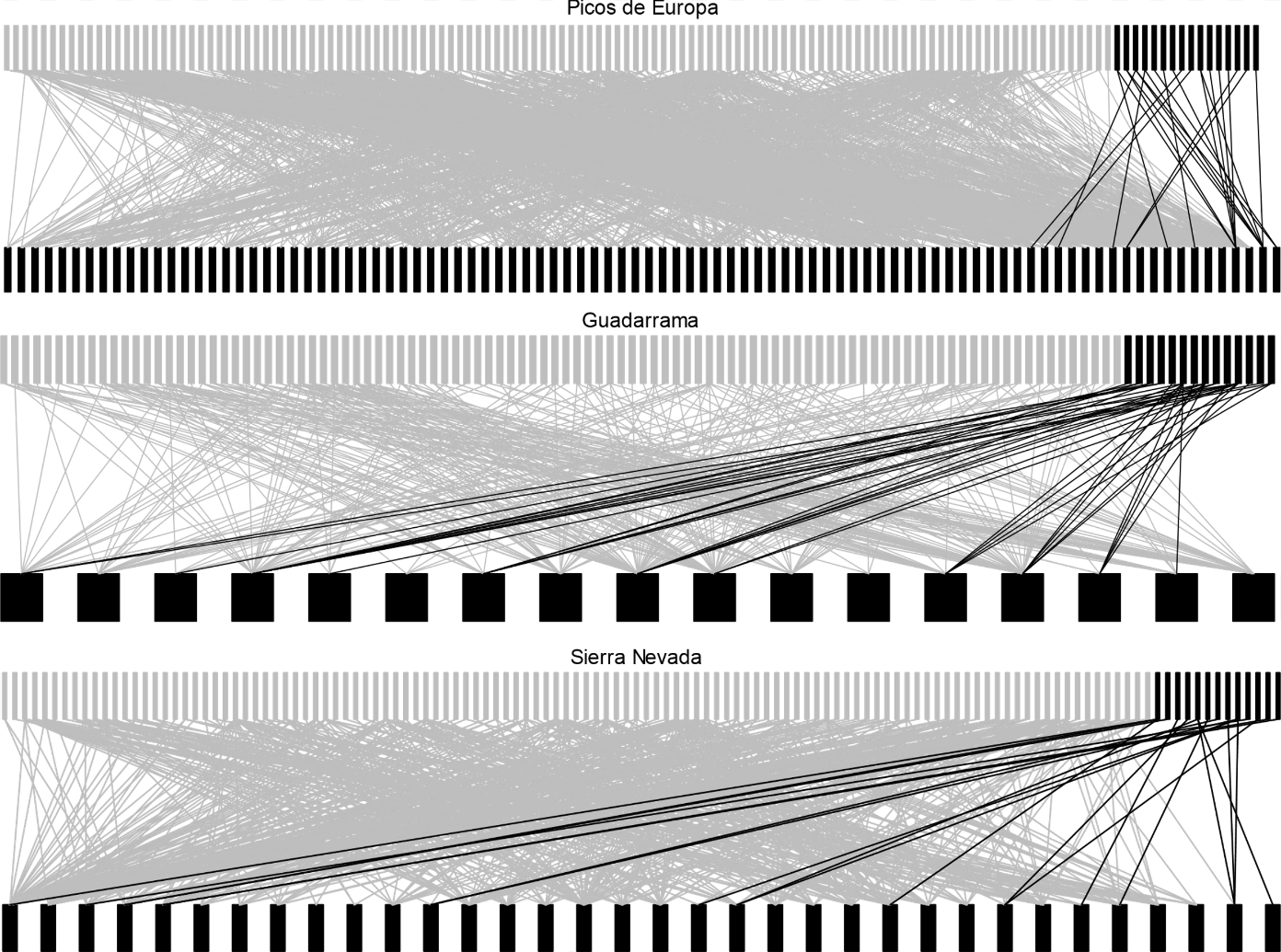


**Figure S1** Combined qualitative plant-flower visitor networks for the three studied high mountain sites. Within each network, boxes in the upper row represent animal species and boxes in the lower row plant species. Diurnal insects and their interactions are shown in grey and nocturnal insects and their interactions in black.

Appendix S3. Sampling completeness.

**Table S4.** Interaction sampling completeness (%) in the three study sites based on the number of pollinator visits for diurnal networks, and on nocturnal moths with pollen attached for nocturnal networks. O: observed interactions, E: estimated interactions.

|  | Diurnal | | |  | Nocturnal | | |
| --- | --- | --- | --- | --- | --- | --- | --- |
| Site | % | O | E |  | % | O | E |
| Picos de Europa | 72 | 1136 | 1588 |  | 36 | 10 | 29 |
| Guadarrama | 75 | 315 | 419 |  | 28 | 37 | 134 |
| Sierra Nevada | 80 | 546 | 683 |  | 8 | 19 | 247 |

**Table S5.** Species sampling completeness (%) in the three study sites based on the number of links detected in diurnal, octurnal and combined networks. A: animal species, V: plant species.

|  | Diurnal | |  | Nocturnal | |  | Combined | |
| --- | --- | --- | --- | --- | --- | --- | --- | --- |
| Site | A | V |  | A | V |  | A | V |
| Picos de Europa | 97 | 92 |  | 64 | 75 |  | 96 | 88 |
| Guadarrama | 69 | 100 |  | 76 | 83 |  | 70 | 100 |
| Sierra Nevada | 62 | 99 |  | 23 | 57 |  | 58 | 100 |
